# Graves’ orbitopathy occurs sex-independently in an autoimmune hyperthyroid mouse model

**DOI:** 10.1038/s41598-018-31253-4

**Published:** 2018-08-30

**Authors:** Anke Schlüter, Ulrich Flögel, Salvador Diaz-Cano, Gina-Eva Görtz, Kerstin Stähr, Michael Oeverhaus, Svenja Plöhn, Stefan Mattheis, Lars C. Moeller, Stephan Lang, Nikolaos E. Bechrakis, J. Paul Banga, Anja Eckstein, Utta Berchner-Pfannschmidt

**Affiliations:** 1 Molecular Ophthalmology, Department of Ophthalmology, University of Duisburg-Essen, Essen, Germany; 2 Department of Oto-Rhino-Laryngology, Head and Neck Surgery, University Hospital Essen, Essen, Germany; 3 Experimental Cardiovascular Imaging, Department of Molecular Cardiology, Heinrich Heine University Düsseldorf, Düsseldorf, Germany; 4 Faculty of Life Sciences & Medicine, King’s College London; King’s College Hospital NHS Foundation Trust, London, United Kingdom; 5 Department of Ophthalmology, University Hospital Essen, Essen, Germany; 6 Department of Endocrinology, Diabetes and Metabolism, University Hospital Essen, Essen, Germany

## Abstract

Graves’ orbitopathy (GO) is the most common extra thyroidal complication of Graves’ disease (GD) and occurs predominantly in women but more severe in men. The reason for this effect of gender on GO is unknown. Herein we studied the manifestation of GO in both sexes of an induced mouse model in absence of additional risk factors present in patients like advanced age, genetic variabilities or smoking. Male and female mice were immunized with human TSHR A-subunit encoding plasmid. Both sexes comparably developed autoimmune hyperthyroidism characterized by TSHR stimulating autoantibodies, elevated T4 values, hyperplastic thyroids and hearts. Autoimmune mice developed inflammatory eye symptoms and proptosis, although males earlier than females. Serial *in vivo* ^1^H/^19^F-magnetic resonance imaging revealed elevated inflammatory infiltration, increased fat volume and glycosaminoglycan deposition in orbits of both sexes but most significantly in female mice. Histologically, infiltration of T-cells, extension of brown fat and overall collagen deposition were characteristics of GO in male mice. In contrast, female mice developed predominately macrophage infiltration in muscle and connective tissue, and muscle hypertrophy. Apart from sex-dependent variabilities in pathogenesis, disease classification revealed minor sex-differences in incidence and total outcome. In conclusion, sex does not predispose for autoimmune hyperthyroidism and associated GO.

## Introduction

Graves’ disease (GD) is an autoimmune thytoid disorder that is caused by antibodies directed against the TSH-receptor (TSHR) leading to hyperthyroidism. In 30–50% of GD patients overt orbitopathy occurs, termed Graves’ orbitopathy (GO) or thyroid eye diseases^1^. GO symptoms are variable and depend on extend of orbital tissue inflammation and edema, expansion of adipose tissue and muscle fibrosis, resulting in disfiguring forward protrusion of the eye (proptosis) and/or muscle dysfunction (squint)^2,3^. The eye disease significantly impairs quality of life, may be sight-threatening and limited therapeutic options are available^4,5^. There is an urgend need for disease prevention or even early treatment, but confounding factors underlay the difficulty as the condition has a multifactorial etiology where no single factor predict the clinical outcome^6–8^. Manifestation of GO in GD patients is correlated with elevated serum concentrations of TSHR antibodies and severe hyperthyroidism^9,10^. Risk factors for development or worsening of GO are gender, age and multiple genetic variabilities in combination with exogenous factors like smoking, stress, infections or iodine intake^6,7^. Of note, among risk factors, female gender appears to conferr a greater risk than any other single factor for development of GO where depending on the different populations examined GO is found with female-to-male ratio of 4 to 6^11–14^. However, female-bias depend on severity of GO: The female to male ratio was 9.3 in patients with mild orbitopathy, 3.2 in those with moderate orbitopathy and 1.4 with severe orbitopathy^15^. Altogether the eye disease tends to be more common in younger woman but more severe in men and older patients^15–18^. The reason underlying gender as a predisposing factor is unclear although, hormones and sex linked genetic susceptibility may be major factors. Moreover, the gender-specific impact of additional risk factors like advanced age and smoking on GO remains unclear and could not yet be investigated experimentally because of a lack of a male mouse model for GD and associated GO.

Experimental mouse models of autoimmune hyperthyroidism have been reported where *in vivo* expression of the extracellular region of the human TSHR (termed hTSHR A-subunit) by genetic immunizytion leads to the induction of experimental GD in the female model^19–21^. Recently we reported on the development of a robust and reproducible model of experimental autoimmune hyperthyroidism with associated GO in female BALB/c mice immunized by electroporation with a plasmid encoding hTSHR A-subunit and more recently, further shown that by this method we can disturb the normal mechanism of immune tolerance by inducing disease with self-anitgen, mouse TSHR in the model^22–24^. The female GO mouse model induced with hTSHR A-subunit closely resembles GO of patients sharings many pathological features with the condition. Importantly, different animals undergoing experimental GO show disease heterogeneity with varying degrees of orbital muscle inflammation and extension of adipose tissue, similar to that observed in patients with GO^22,23,25^.

In the present study, we aimed to elucidate the impact of gender in the experimental GO mouse model in absence of additional risk factors. We show that immunization with the hTSHR A-subunit plasmid induced auto-antibodies and leads to comparable manifestation of autoimmune hyperthyroidism and orbitopathy in male mice as well as in females. Our study demonstrates sex-dependent variabilities in onset and progression of orbitopathy rather than female-biased prevalence and incidence of disease. Our study suggests a minor contribution of sex on its own for manifestation of GO. However, additional risk factors linked to gender in patients most likely genetic variabilities, advanced age and/or smoking may be major determinants for development of substantial female-bias in GO.

## Results

### Effect of sex on auto-immune reactivity in GO mouse model

**Figure 1:**
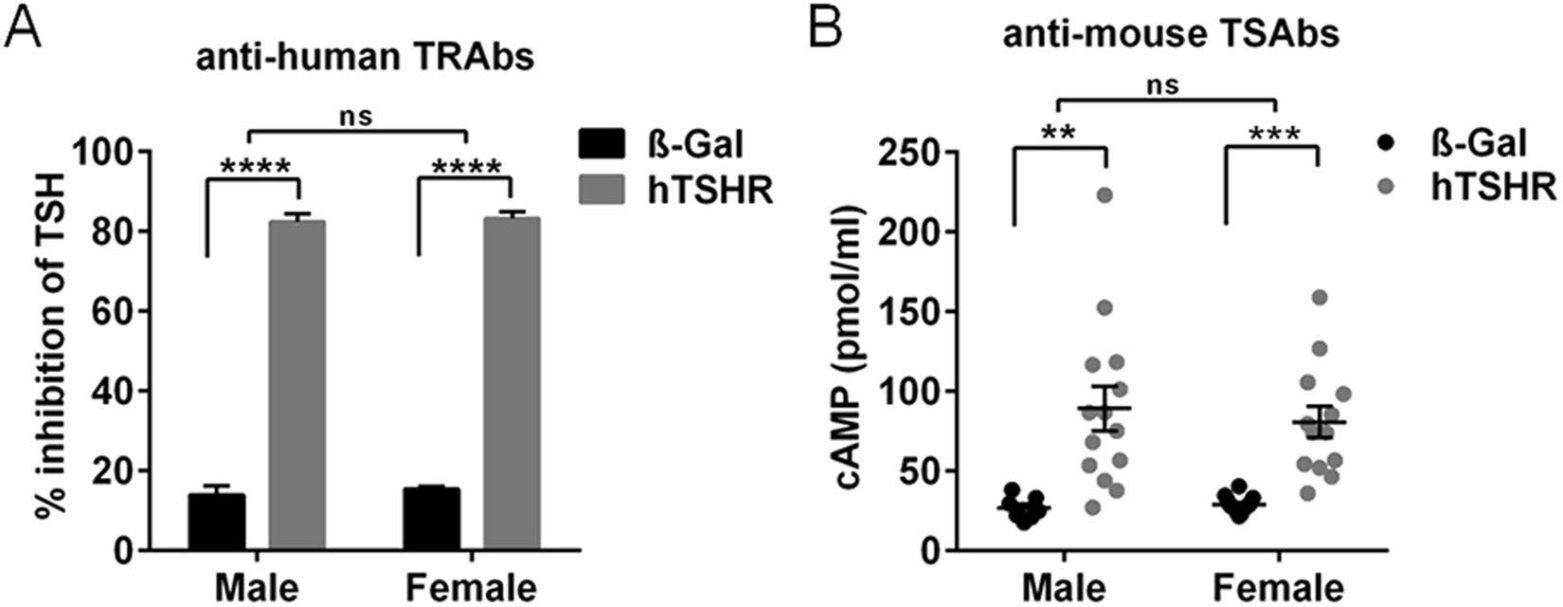
Evaluation of TSHR antibodies in GO mouse model. Total TSHR antibodies and stimulating activity of antibodies was measured in sera of individual male or female mice immunized either with ß-Gal (males n = 8, females n = 16) or hTSHR A-subunit plasmid (males n = 14; females n = 14). (**A**) Total TSHR antibodies (TRAbs) were measured by TSH binding inhibitory immunoglobulin activity to the human TSHR given as % inhibition of TSH. (**B**) Stimulating activity of antibodies (TSAbs) was evaluated by bioassay using CHO cells stably expressing the mouse TSHR. Stimulating activity is given in cAMP (pmol/ml). Statistical significance is indicated ****p ≤ 0.0001, ***p ≤ 0.001, **p ≤ 0.01, ns not significant.

To investigate whether immunization with hTSHR A-subunit encoding plasmid has led to an immune reaction we evaluated total TSHR antibodies (TRAbs) in the mice sera by measuring TSH binding inhibitory immunoglobulin values in competition with labeled bTSH to human TSHR. 100% of GO mice of both sexes equally developed TRAbs with an inhibition activity range of 80% inhibition of TSH binding (Fig. 1A). To evaluate auto-immunoreactivity to the mouse TSHR, we measured the cAMP production of CHO cells stably transfected with mouse TSHR in response to the sera (Fig. 1B). 90% of both sexes exhibited TSHR antibodies with stimulating activity (TSAbs) to the mouse TSHR when compared to the respective ß-Gal immunized control mice (Fig. 1B). The results strongly indicate that male and female BALB/c mice are equally susceptible to immunization with hTSHR A-subunit plasmid as both sexes showed comparable (auto-) immunoreactivity and developed similar high levels of TSHR antibodies with stimulating activity to self-antigen.

### Alterations in thyroid function in both sexes of GO mouse model

**Figure 2:**
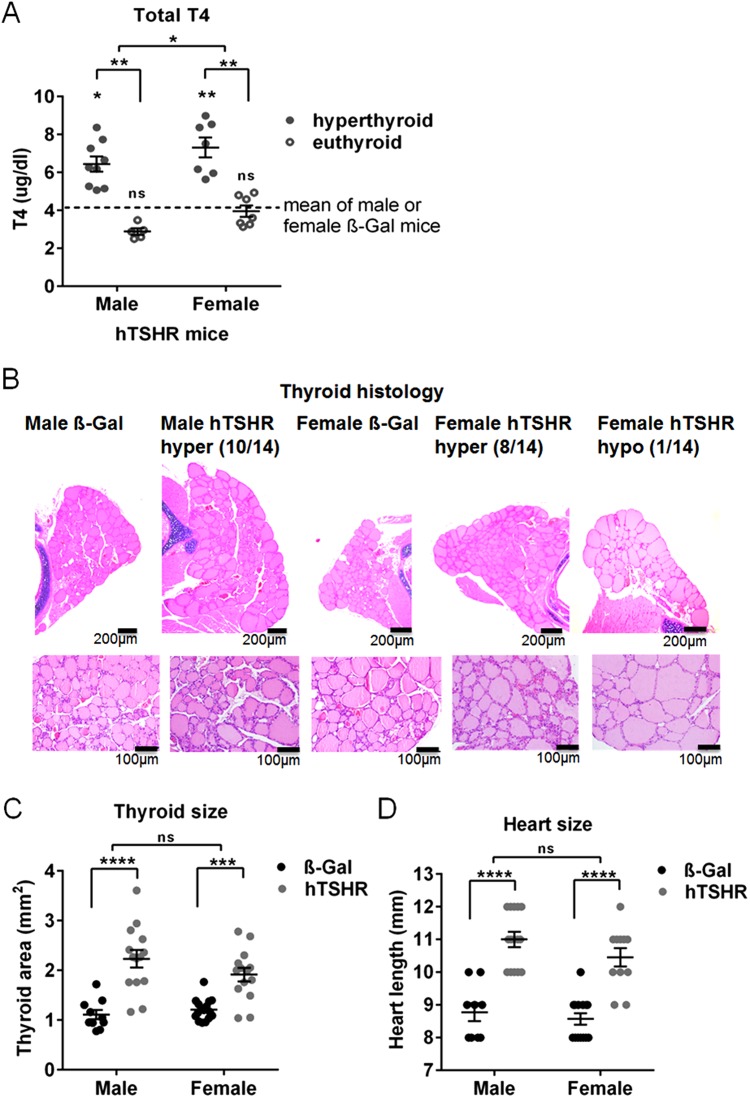
Evaluation of thyroid function and morphology. (**A**) Total T4 values (µg/dl) were measured by ELISA in serum of male or female ß-Gal (males n = 8, females n = 16) or hTSHR mice (males n = 14, females n = 14). Dotted lines show the mean values obtained from male (4.17 ± 0.79) or female ß-Gal control mice group (4.19 ± 0.33). Male or female hTSHR mice with statistically elevated serum T4 values (hyperthyroid) or unchanged values (euthyroid) compared to euthyroid ß-Gal control mice are shown. Statistical significance is indicated, **p ≤ 0.001, *p ≤ 0.05. (**B**) Thyroid slices of the animals were H&E stained and thyroid state was evaluated (ß-Gal males n = 10, females n = 16; hTSHR males n = 14, females n = 14). Representative images of thyroids are shown, upper panel magnification x4, lower panel magnification x20. Number of hyperactive (hyper) or hypoactive (hypo) indexed mice are given. Hyperactive thyroids were characterized by increase of total thyroid size, cuboid cylindrical follicular cells with small amount of colloid, thick follicular epithelium. Hypoactive thyroids showed thin follicular epithelium and in some follicles the follicular membrane was almost not visible. For high-magnification pictures see also S1 Fig. (**C**) Thyroid area was measured in H&E stained thyroid slices and is expressed in mm^2^. (**D**) Heart length of individual animals was measured and is expressed in mm (ß-Gal males n = 9, females n = 14; hTSHR males n = 14, females n = 11). Statistical significance is indicated ****p ≤ 0.0001, ***p ≤ 0.001, ns not significant.

To investigate whether thyroid function of the mice was altered total T4 was measured in serum (Fig. 2). Statistically significant elevation of total T4 values indicating hyperthyroid hormone status were observed in 9/14 (64.3%) male and 7/14 (50%) female hTSHR mice, all other mice remained normal euthyrroid compared to the respective ß-Gal control mice group (Fig. 2A). Morphologically 10/14 male and 8/14 female hTSHR mice showed stimulated thyroid follicles characterized by cuboid cylindrical follicular cells with a small amount of colloid indicating hyperactivity (Fig. 2B). Thyroid morphology of one female GO mouse showed flat follicular epithelium indicating hypoactivity. Thyroid morphology of all other mice remained normal similar to ß-Gal control mice. There was no evidence of inflammatory cell infiltration in any of the thyroid glands (Fig. 2B). As a consequence of hyperthyroidism thyroid and cardiac hypertrophy has been described in female mice to develop GD after prolonged hTSHR A-subunit immunization^26^. Likerwise we observed a statistically significant increase in the thyroid area as well as heart length in hTSHR mice compared to the corresponding ß-Gal mice indicating thyroid and cardiac hypertrophy in both sexes (Fig. 2C,D). Weight of animals remained unaffected, although male mice weighted more (ß-Gal 25.8 ± 0.5; hTSHR 26.8 ± 0.3) when compared to the females (ß-Gal 22.9 ± 0.4; hTSHR 23.9 ± 0.4). The results suggested that immunization with the hTSHR A-subunit encoding plasmid led a comparable portion of male or female mice to develop autoimmune hyperthyroidism.

### Overt eye symptoms indicating orbitopathy in mice of both sexes

**Figure 3:**
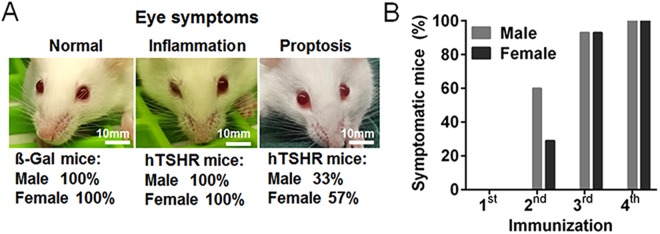
Inspection of mice eye symptoms indicating orbital disease. During the entire experimental course the mice were individually inspected for eye signs indicating orbital disease (ß-Gal males n = 10, females n = 17; hTSHR males n = 15, females n = 14). (**A**) Representative images of a ß-Gal mouse showing any pathological eye signs (left) and of hTSHR mice with acute symptoms of inflammation like red canthus and pus (middle) or with proptosis (right). Percentage of male or female mice showing eye symptoms is indicated. (**B**) Percentage of hTSHR mice showing eye symptoms during the course of immunizations (Symptomatic mice). Eye signs were monitored during the three weeks between the immunizations and after the 4th immunization before scarifying the animals. On the x axis the time points are defined as after the 1st, second, third and fourth immunization.

During the entire experimental course the mice were individually inspected for clinical eye symptoms indicating orbital disease (Fig. 3). ß-Gal control mice did not show any GO typical changes, while 100% male and female hTSHR mice showed eye signs of acute inflammation like redness, pus and eye lid swelling. Additionally, 33% of the males and 57% of female GO mice showed proptosis (males: 5/15 animals; females: 8/14 animals) (Fig. 3A). Inflammatory eye symptoms appeared transiently asymmetric with both eyes affected by the entire experimental course while proptosis appeared symmetric. The earliest inflammatory eye symptoms were found in hTSHR mice after the second immunization step, when 53% of the males and 29% of the females presented eye signs (males: 8/15 animals; females: 4/14 animals). Most mice of both sexes developed eye signs after the third immunization step (males: 14/15; females: 13/14) and finally until 6 weeks after the last immunization, all of the hTSHR mice have had developed symptoms of both eyes indicating inflammation or soft tissue changes (Fig. 3B).

### MRI of orbital disease in living male vs female GO mice

**Figure 4:**
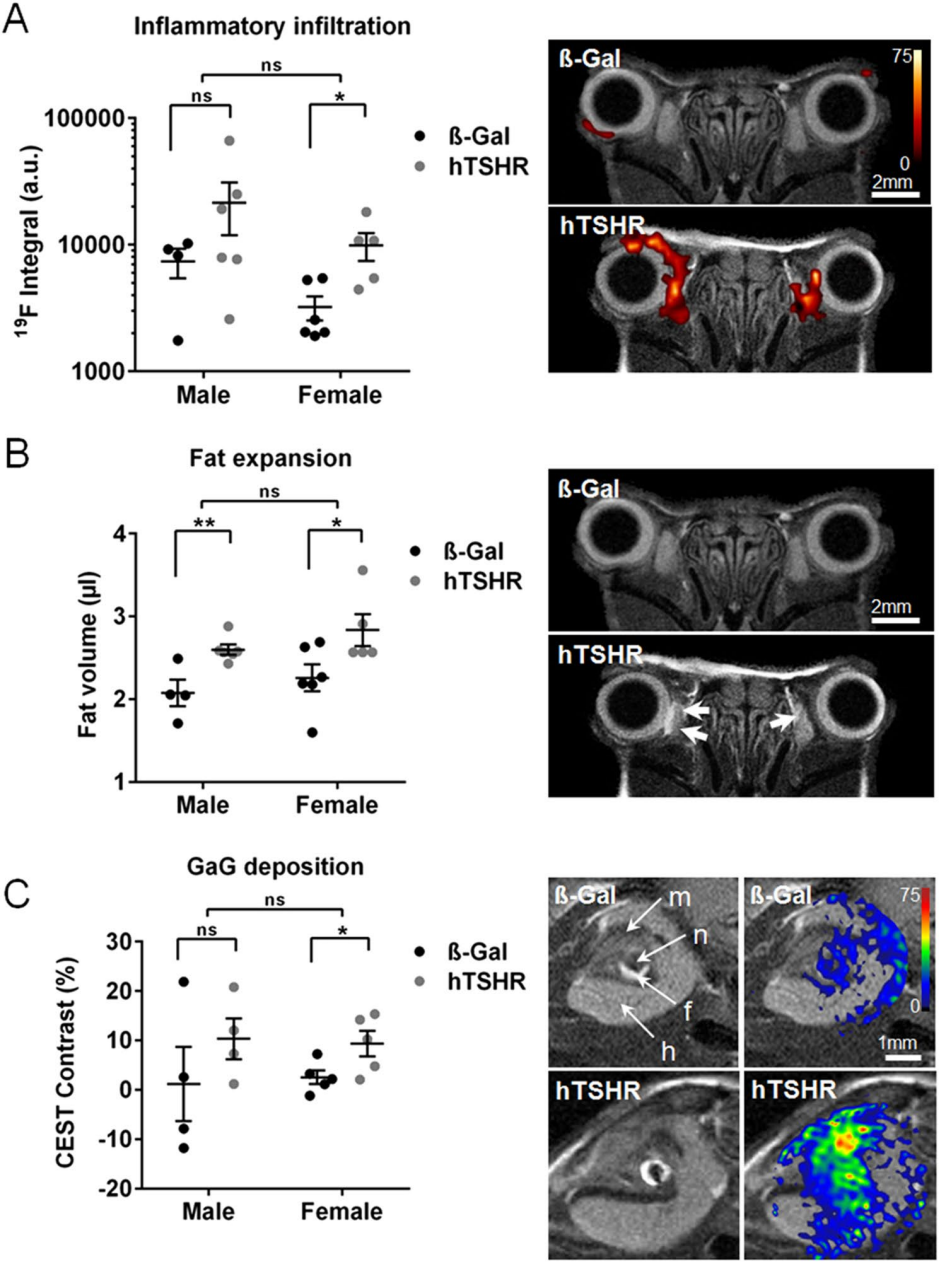
Detection of key features of orbitopathy by MRI in living mice. Six weeks after the last immunization mice each group (ß-Gal males n = 4, females n = 6; hTSHR males n = 6, females n = 5) were injected with PFC and subjected to serial ^1^H/^19^F MRI. Several ^1^H/^19^F MRI scans of individual mice were quantificated. Representative MRI images of a male ß-Gal and hTSHR mice are shown. (**A**) ^19^F integral of PFC-loaded macrophages/monocytes are expressed in arbitrary units (a.u.). Overlay of corresponding anatomical ^1^H and ^19^F MRI images showing massive infiltration of PFC-loaded monocytes/macrophages in orbital tissue. ^19^F intensity is shown in false color as indicated. (**B**) T2-weighted ^1^H MRI. Orbital fat volume is given in (µl). Axial sections are showing expansion of fat tissue in the orbit as indicated by arrows. (**C**) T2-weighted ^1^H MRI. ^1^H CEST contrast of glycosaminoglycans (GAGs) is expressed in %. Three mice showing a weak image quality has been excluded from the CEST analysis; data shown from ß-Gal males n = 4, females n = 4 and hTSHR males n = 5, females n = 5. Oblique sections are showing orbital tissues as indicated (m, muscles; n, nerve; f, fat tissue; h, haderian gland). Overlay of anatomical images and corresponding CEST signal indicating deposition of GAGs. CEST signal is shown in false color as indicated. Statistical significance is indicated **p ≤ 0.01, *p ≤ 0.05, ns not significant.

To follow inflammatory events and remodeling of orbital tissue *in vivo* we performed serial non-invasive MRI in living mice (Fig. 4). Six weeks after the last immunization 4–6 male or female mice randomly selected each group were subjected to serial analysis of ^1^H/^19^F MRI procedure. T2-weighted MRI covering the entire orbit to image the distinct tissue types (muscle, adipose tissue, nerve and all anatomical structures) was carried out. ^19^F MRI revealed elevated infiltration of macrophages/monocytes in the peri- and retro- orbital tissue at muscles and nerve tissue of male and female mice (Fig. 4A). Statistically significant inflammation was detected in the female hTSHR mice compared to ß-Gal females (Fig. 4A). Compared to the females the males showed a significant variance in inflammation. Notably, 3 of the five male mice showed a higher abundance of macrophages/monocytes in the orbital tissue than any of the female mice (Fig. 4A). Significantly increased expansion of orbital fat volume could be detected in male as well as female hTSHR mice by ^1^H MRI (Fig. 4B). Furthermore, mice were subjected to ^1^H CEST MRI to detect glycosaminoglycans (GAGs) such as hyaluronic acid in orbital tissues^27^. Quantification of the CEST signal in the retro orbital area including the harderian gland revealed a statistically significant increase in GAG deposition in female hTSHR mice while the males tended to an increase in GAG deposition in comparison to the corresponding ß-Gal mice (Fig. 4C). In summary, critical features of orbitopathy could be efficiently detected in living male as well as female hTSHR mice by the use of combined ^1^H/^19^H MRI. Serial MRI revealed significant inflammation, fat expansion, as well as GAG deposition in orbits of both sexes but most significant in female mice.

### Effect of sex on mouse orbital pathological abnormalities

**Figure 5:**
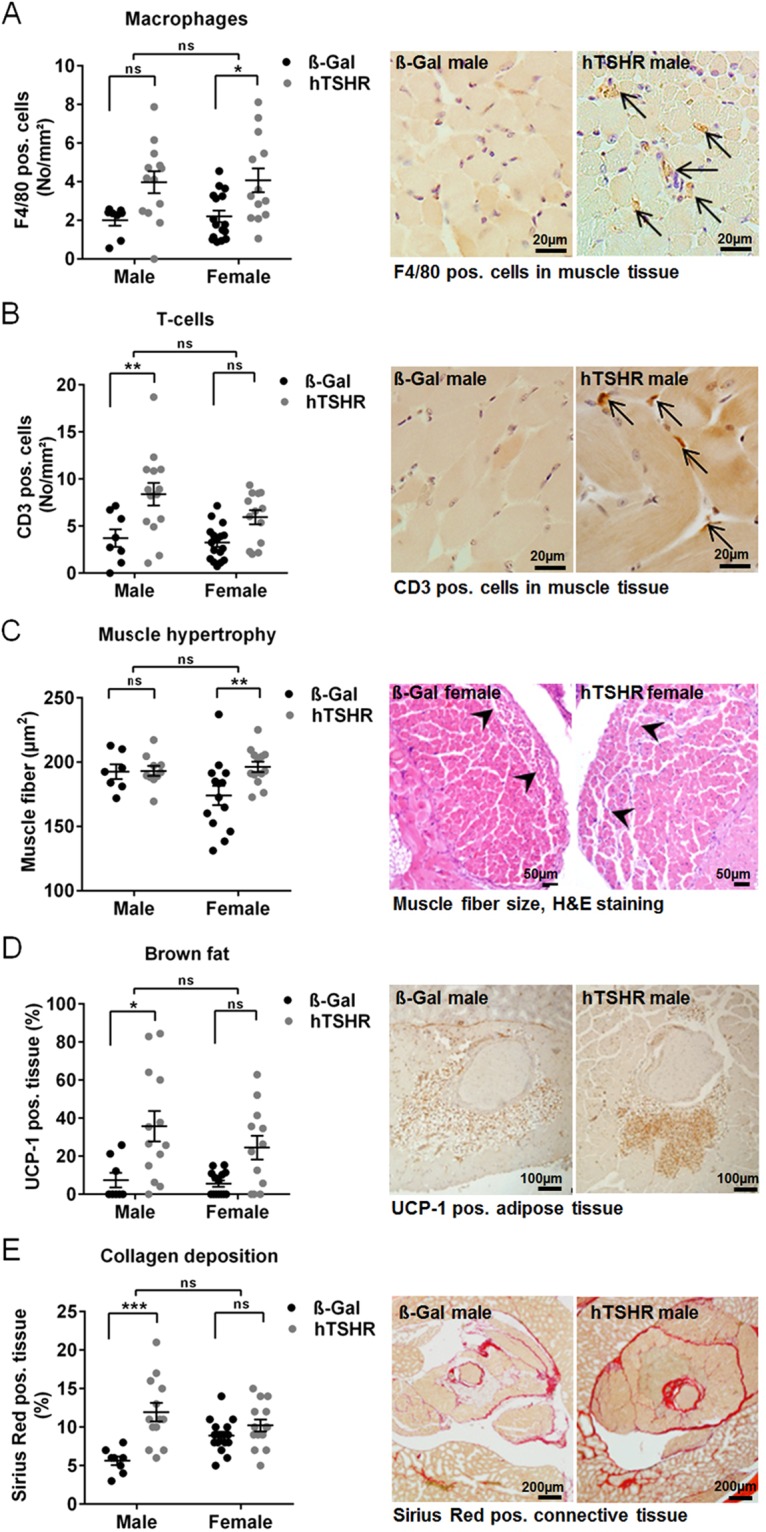
Orbital tissue abnormalities in GO mouse model analyzed histologically. The orbits of mice (ß-Gal males n = 8, females n = 15; hTSHR males n = 14, females n = 14) were fixed, paraffin embedded and consecutive slices of the middle orbital area were subjected to different staining procedures. Representative pictures of stainings are shown respectively, and corresponding high-magnification images are shown in Figs S2–5. The stainings were quantitatively analyzed as described in methods. (**A**,**B**) F4/80 as a marker for macrophages, CD3 as a marker for T cells. Cells positive for F4/80 or CD3 were counted in the extraocular muscle tissue and perineural connective tissue and are given in No/mm^2^. Magnification x40, arrows indicate pos. stained cells. (**C**) H&E staining of slices. The size of muscle fibers in extraocular rectus inferior muscle and medial rectus muscle were quantified and is expressed as muscle fiber (µm^2^). Magnification x20. (**D**) UCP-1 as a marker for brown/beige fat. UCP-1 positive stained fat was quantified and is expressed as UCP-1 positive fat in % of total adipose tissue. Magnification x10. (**E**) Picrosirius red staining to detect collagens. Picrosirius red staining of retro orbital area (perineural connective tissue, adipose tissue and extraocular muscle tissue) was quantified and normalized to total retro orbital area (Sirius red pos. area (%). Magnnification x4. Statistical significance is indicated ***p ≤ 0.001, **p ≤ 0.01, *p ≤ 0.05, ns not significant.

To investigate sex-specific pathological abnormalities in the diseased orbital tissue in more detail, orbits of all mice were histologically analyzed (Figs 5 and S2–5). Consecutive slices of the middle area were stained immunohistochemically for F4/80 (as a macrophage marker) and CD3 (as a T cell marker) to evaluate the presence of inflammatory cells in the orbital tissues. Female hTSHR mice showed a statistically significant elevated abundance of macrophages in the muscle and perineural connective tissue while male mice showed elevated levels of T cells compared to ß-Gal mice groups (Figs 5A,B and S2). We further examined for evidence of diseased orbital muscles in H&E sections by studying inferior rectus and medial rectus muscle in all immunized mice, which are frequently affected in GO patients^28^. Male hTSHR mice did not show any differences whereas female mice showed statistically significant enlarged muscle fibers in comparison to ß-Gal mice indicating muscle hypertrophy (Figs 5C and S3).

Earlier we have found that the retro-orbital fat tissue of several mouse strains contained significant increase in brown fat (BAT) that expressed TSHR to high levels suggesting BAT to be a potential target tissue in mice^24,29^. We selected slices from the middle orbital area for immunohistochemical staining for UCP-1 expression as a marker for BAT (Figs 5D and S4). Statistically significant elevated portions of BAT was present in the total orbital adipose tissue of male hTSHR mice compared to ß-Gal mice suggesting that an increase in BAT contributed to total fat expansion (Fig. 5D). Just like the males, the female hTSHR mice showed an increasing abundance of BAT although statistical significance was not reached (Fig. 5D).

We next analyzed Sirius Red staining of orbital slices to investigate collagen deposition in the retro orbital area compromising adipose, perineural and extraocular muscle tissue (Figs 5E and S5). Most of the male hTSHR mice showed a significant increase in orbital collagen deposition compared to ß-Gal mice whereas female hTSHR mice did not show any differences in collagen deposition (Fig. 5E). Taken together sex-dependent orbital tissue abnormalities can be detected histologically in the GO mouse model. T cell infiltration, extension of brown adipose tissue and overall collagen deposition were characteristics of GO in male mice. In contrast, female mice developed predominately macrophage infiltration and muscle hypertrophy.

### Comparison of total disease incidence and outcome between sexes

**Figure 6:**
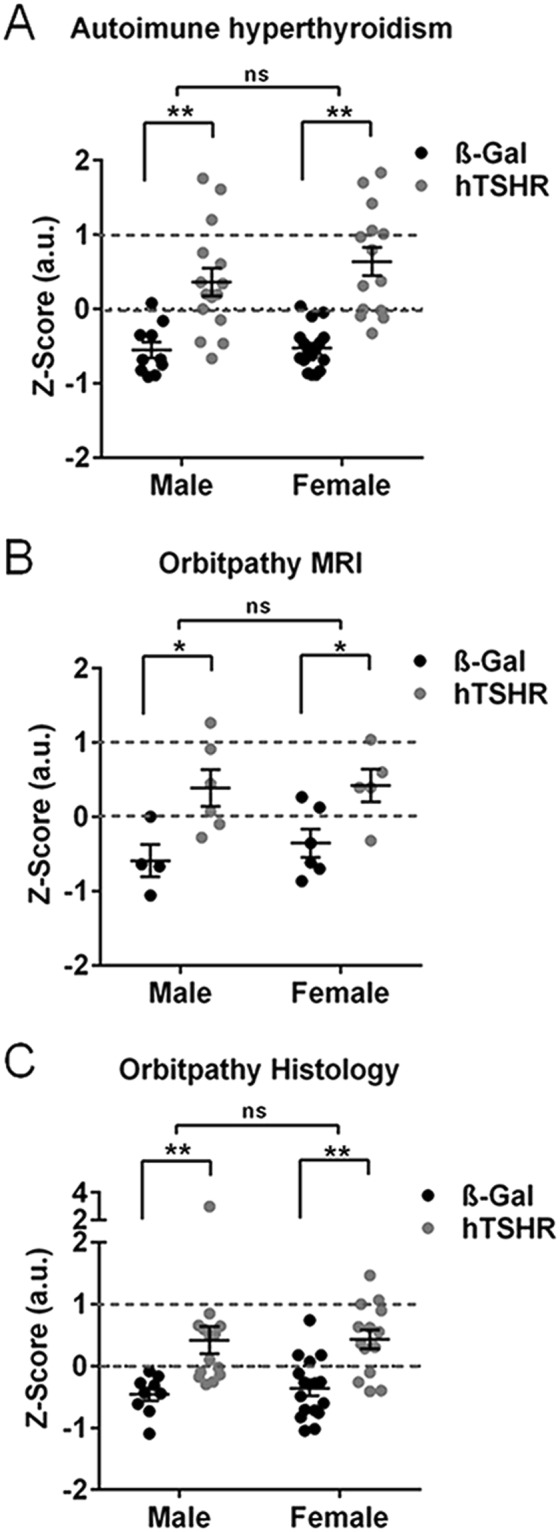
Comparison of male versus female GO mouse model. To compare the total outcome of male versus female autoimmune hyperthyroidism or associated orbitopathy, the characteristic parameters were normalized by the Z-Score method. (**A**) Z-Score of autoimmune hyperthyroidism includes values of TSAbs (Fig. 1), T4 and thyroid area (Fig. 2). (ß-Gal males n = 10, females n = 15; hTSHR males n = 14, females n = 14) (**B**) Z-Score of orbitopathy evaluated by MRI includes parameter inflammatory infiltration, fat extension and GaG deposition (Fig. 4). (ß-Gal males n = 4, females n = 6; hTSHR males n = 6, females n = 5) (**C**) Z-Score of orbitopathy characterized by the histological parameter of, abundance of macropahes, T cells, muscle fiber hypertrophy, BAT extension and collagen deposition (Fig. 5). (ß-Gal males n = 8, females n = 15; hTSHR males n = 14, females n = 14). Statistical significance is given with **p ≤ 0.01, *p ≤ 0.05, ns non-significant.

To compare the different aspects of autoimmune hyperthyroidism and associated orbitopathy at its whole the data sets characterizing autoimmune hyperthyroidism (TSAbs, T4 values and thyroid area) and orbitopathy investigated by MRI (Fat volume, ^19^F Integral, CEST Contrast) or by histology (F4/80, CD3, muscle fiber, UCP-1, collagen) were normalized by using the Z-Score method. The Z-Score of all data sets revealed statistically significant autoimmune hyperthyroidism and orbitopathy in the males, as well as the female GO mouse model to a comparable extent (Fig. 6A–C). Finally we classified the disease outcome along the Z-Score values to evaluate incidence and severity of the disease. Following the clinical activity score of GO patients we categorized clinical disease of mice in mild and moderate-to-severe disease (Table 1). The incidence of clinical autoimmune hyperthyroidism was comparable in both sexes (male: 71%, females 79% of animals). Likewise incidence for clinical GO evaluated was comparable in both sexes. Herein the two techniques of MRI (males: 67%, females: 80%) and histology (males: 64%, females: 71%) gave comparable results. However, moderate-to-severe autoimmune hyperthyroidism as well as orbitopathy was more frequently developed by female hTSHR mice compared to males (Table 1).

**Table 1:** Classification of autoimmune hyperthyroidism and Graves’ orbitopathy in male versus female GO mouse model.

Disease classification	Z-Score	Males		Females	
		No	%	No	%
**Autoimmune hyperthyroidism**					
Subclinical	≤0	4	**28.6**	3	**21.4**
Clinical	>0	10	**71.4**	11	**78.6**
Mild	>0–1	7	50.0	4	28.5
Moderate-to-severe	>1	3	21.4	5	35.7
**Graves’ orbitopathy MRI**					
Subclinical	≤0	2	**33.3**	1	**20.0**
Clinical	>1	4	**66.7**	4	**80.0**
Mild	>0–1	3	50.0	3	60.0
Moderate-to-severe	>1	1	16.7	1	20.0
**Graves’ orbitopathy Histology**					
Subclinical	≤0	5	**35.7**	4	**28.6**
Clinical	>0	9	**64.3**	10	**71.4**
Mild	>0–1	8	57.1	7	50.0
Moderate-to-severe	>1	1	14.3	3	21.4

Disease classification was done along the Z-score values in Fig. 6. Number of mice is given in (No) or %. Subclinical disease (Z-score < 0): Finally these mice did not significantly manifest autoimmune hyperthyroidism and/or GO although these hTSHR A-subunit immunized mice developed TSHR autoantibodies and/or transient inflammatory eye symptoms. Clinical disease (Z-score > 0): These mice significantly manifested chronical disease during the experimental course. Clinical disease is classified in mild and moderate/severe in accordance with the Z-score values as indicated (mild: Z-score > 0–1; moderate/sever: Z-score > 1).

## Discussion

### Conflicting data on impact of sex in mouse models in Graves’ disease and orbitopathy

Several experimental mouse models of GD have been developed in female mice and among several mice strains BALB/c have been found most susceptible to different immunization methods^30^. In the present study we have successfully established a male mouse model of autoimmune hyperthyroidism associated orbitopathy by immunisation with the hTSHR A-subunit coding plasmid and compared it to the corresponding female model. Our results strongly indicate that male and female BALB/c mice are equally susceptible to the immunization method and showed comparable auto-immunoreactivity as well as thyroid and orbital pathology. Regarding thyroid disease we show a lack of substantial female-bias just like reported in earlier studies of GD models using inbred mice strains AKR/N or BALB/c^19,31^. In contrast, Costagliola *et al*. (2000) reported a substantial female-bias in outbred NMRI mice immunized with hTSHR holoreceptor cDNA^32^. Although incidence of disease remained low the study might indicate that the use of an outbred mice strain is in favor of female-bias in induced GD mouse models. However, in mouse models of other autoimmune diseases, in induced systemic lupus erythematosus and experimental autoimmune encephalomyelitis (EAE) female-bias existed in inbred SJL mice^33^. In contrast, male B10.PL and PL/J mice had more susceptibility to EAE than females while no sex-bias induced in NOD and C57BL/6 mice^33^. The presence of female-bias in one inbred mouse strain, but not another, indicates that genetic background of the mice can influence sex differences in experimental autoimmunity. In addition also different approaches to develop TSHR autoimmunity in animals may effect the sex-specific outcome. In a transgenic hTSHR/NOD.H2^h4^ mouse model female mice were shown to be more prone than male mice in spontaneously developing TSHR antibodies and elevated T4 levels^34^. Although some of the females exhibited elevated T4 levels none of the thyroid glands showed signs of hypertrophy as typically observed in induced models of GD (24). Compared to earlier studies addressing the influence of sex in GD models our study demonstrate the incidence of TSAbs (90% of animals), hyperthyroidism (50–70% of animals) and orbitopathy (60–70% of animals) significantly improved by hTSHR A-subunit immunization of BALB/c mice in both sexes.

### Effect of sex on immune-reactivity and prevalence in Graves’ orbitopathy

Sex differences exist in the animal model regarding disease onset during the experimental course. Male mice develop acute inflammatory eye symptoms earlier than female mice indicating a faster response toward the immunization. Although in this study we gained little information on the TSHR antibody level at this early time point of disease induction it is likely that the immune male mice underwent a faster and stronger immune-response initially. However, at the end stage, the TSHR antibody levels were comparable in both sexes suggesting similar immune-reactivity while ongoing disease.

In general female-bias in autoimmunity is explained by stronger humoral and cellular immune responses of females due to sex hormone and x-chromosomal dependent mechanisms^35^. However, sex-based immune mechanisms leading to female-bias in thyroid autoimmunity are poorly understood^36–39^. It appears that potential sex dependent mechanisms which would have caused female-bias in autoimmunity did not take effects in the GO mouse model. These finding indicates that either endogenous and/or exogenous risk factors additionally linked to gender are most likely critical determinants for female-bias in thyroid autoimmunity and GO rather than sex on its own. Potential endogenous risk factors absent in the mouse model which could have predisposed female-bias in GO are most likely advanced age in conjunction with susceptibility genes and (epi-) genetic variations. Genetic predisposition for GO is believed to overlap with Graves’ hyperthyroidism^40^. Immune response modulating genes HLH DR3, CTLA4, FOXP3, CD40L, and thyroid specific genes TSHR has been suggested to predispose for GD and GO^41–43^. Also associations between polymorphysms in immunological factors like INFγ, TNFα or ICAM1 and GO were suggested. However, the reported associations vary between populations and lacked associations with gender^44,45^. Furthermore exogenous risk factors may linked to gender in GO like smoking or infection which could have influenced immunity have been absent in the mouse model^46–48^. On the other hand female-bias in GO patients might be overdiagnosed due to gender differences in behavior of the health system, which is of course, absent in the model^49^.

### Sex differences in progression of pathological orbital tissue abnormalities

Sex differences exist regarding the progression of pathological tissue abnormalities. Male mice developed acute inflammatory eye signs earlier which finally resulted in extensive infiltration of inflammatory cells in the orbit. Inflammation was restricted to the muscle and perineural tissue in both sexes, but males did not show any muscle hypertrophy like the females. Muscle hypertrophy appeared to be a characteristic of female mice since female mice immunized with homologous mouse TSHR A-subunit developed muscle hypertrophy as well^24^. Instead most of the immunized male mice established overall collagen deposition indicating chronical fibrotic changes have already set in the orbital tissue. The results suggest that in comparison to the females the male GO mice started earlier acute inflammation and underwent faster progression into a chronic state of GO. However, apparent proptosis was observed only in 33% of the male but in 57% of the female hTSHR mice. Interestingly orbital fat expansion and browning where comparable in both sexes suggesting that muscle hypertrophy and GAG deposition contributed to proptosis in females. However, simple visual assessment of pathological eye changes may be liable to subjective judgement and histological evaluation of middle area slices of the orbit may not reflect the total orbital disease. To overcome these limitations we exploited small animal MRI to evaluate pathological features of GO in the whole orbita of living mice. Moreover, we scored distinct orbital abnormalities in individual mice to enable comparative analysis of total orbital disease between sexes.

## Conclusions

In the mouse model incidence and severity of autoimmune hyperthyroidism, as well as orbitopathy, was comparable in both sexes although some sex differences existed in onset and progression of pathological features. In conclusion, sex on its own is not a predisposing factor for development or worsening of GO. Additional risk factors linked to sex are likely most important determinants for deveopement of substantial sex differences. The GO mouse model provides a preclinical murine model for autoimmune hyperthyroid associated orbitopathy in both sexes with the opportunity to dissect the impact of critical endogenous or exogenous risk factors as well as treatments of orbital pathology in future studies.

## Materials and Methods

### Mice procedures

Male and female inbred BALB/c OlaHsd (H2d) mice were purchased from commercial supplier Envigo GmbH Netherlands and housed in temperature (23 ± 1 °C) and light –controlled (inverse 12:12 light-dark cycle) conditions. Each four male or female mice were maintained in single ventilated cages in specific pathogen free conditions to avoid environmental exposure. Drinking water and food pellets were provided ad libitum. Mice were immunized at the age of 6–8 weeks and sacrificed at the age of 21–23 weeks. All animal procedures were reviewed and approved by North Rhine Westphalian State Agency for Nature, Environment and Consumer Protection (LANUV), Germany. We confirm that all experiments were performed in accordance with relevant guidelines and regulations. The eukaryotic expression plasmid, pTriEx1.1Neo-human (h)TSHR A-subunit (also known as hTSHR289) and the control pTri1Ex1.1Neo-ß-Gal plasmid were used and referred to as hTSHR A-subunit plasmid and ß-Gal plasmid respectively^22,23^. Briefly, mice were anesthetized with isoflurane and immunized by injection and electroporation of 50 µg (1 mg/ml) plasmid into each biceps femoris muscle four times three weeks apart. A total of 15 male mice and 14 female were immunized with hTSHR A-subunit plasmid (referred as hTSHR mice), ten male and 17 female mice were immunized with control ß-Gal plasmid (referred as ß-Gal mice). All animals were monitored daily for general conditions, spontaneous behavior and clinical outcome of orbitopthy. Mice were inspected daily for eye symptoms indicating orbital disease by two different observers during the entire experimental course. Inflammatory eye symptoms like red eyes and lids, swelling of the lid and upcoming pus indicated early acute disease. Persistent eye symptoms like swelling, pus and proptosis for at least 5 days indicated chronic disease. Squint like eye signs were not observed in any mouse. Mouse weights were measured weekly. Mice were sacrificed when all hTSHR mice have shown eye symptoms six weeks after the last immunization. After scarification serum was obtained from inferior vena cava puncture. Microsurgical excision of thyroid glands and orbits for histology was performed. Hearts were eviscerated and length was measured. One male hTSHR mouse died before sacrification for unknown reasons.

### Small animal magnetic resonance imaging of living mice

Magnetic resonance imaging (MRI) was performed six weeks after the last immunization immediately before sacrificing the mice. Four to six mice per group were randomly selected for MRI. At least 48 h before MRI analysis the animals were injected i.v. with 300 µl perfluorocarbon nanoemulsion (PFC) for *in situ* labeling of macrophages/monocytes and subsequent tracking by ^19^F MRI. Combined ^1^H/^19^F MRI was carried out at a Bruker AVANCEIII 9.4 T wide bore NMR spectrometer driven by ParaVision 5.1 (Bruker, Rheinstetten, Germany). Images were acquired using the Bruker microimaging unit Micro 2.5 with actively shielded gradient sets (1.5 T/m) and a 25 mm birdcage resonator tunable to both ^1^H and ^19^F as described previously^50^. Mice were anaesthetized with 1.5% isoflurane and kept at 37 °C. T2 mapping was carried out using a multi-slice multi-echo (MSME) sequence covering the entire orbit for different tissue types essentially as reported^51^. Chemical exchange saturation transfer (CEST) was performed to analyze glycosaminoglycans (GaGs)^52^. After the acquisition of all ^1^H datasets, the resonator was tuned to ^19^F, and morphologically matching ^19^F images were recorded. For superimposing the images of both nuclei, the “hot iron” color look-up table provided by ParaVision was applied to ^19^F images. To fade out the background noise from ^19^F images a constant threshold was applied to ^19^F data. Using the ROI tool of ParaVision, inflamed regions were determined from ^19^F images by planimetric analysis of PFC signals of both eyes. The full experimental protocol took around 60–90 min, vital functions were supervised during the whole process, which was well tolerated by all mice. For a detailed description of MRI procedures refer to^27^.

### Serological analysis

Anti-TSHR antibodies (TRAbs) and their subtypes stimulating (TSAbs) and total T4 were evaluated in mouse serum as described before^22,23^. Briefly, TRAbs were measured in commercial TRAK kits using 25 µl serum plus 75 µl human control serum as TBII activity in competition with labeled bTSH to the human TSHR (ThermoFisher, BRAHMS, Germany). TSAbs were measured in 3 µl serum plus 147 µl buffer in a bioassay using stably transfected mouse TSHR-CHO cells kindly provided by Sandra McLachlan as described before^24,53,54^. The concentration of TSAbs is directly correlated to the cAMP production of the cells. cAMP concentration in 100 µl of the mouse TSHR-CHO cells supernatants was measured by ELISA (Enzo, Farmingdale, New York USA). Total T4 concentrations were measured in 25 µl serum by ELISA (DRG, Springfield, New Jersey USA).

### Histopathology and immunohistochemistry orbits and thyroids

The orbits were formalin fixed and paraffin embedded. Orbital slices (1 µm) at the anterior, middle and posterior area of the mouse orbits were H&E stained and examined. Consecutive slices of the middle area were selected and subjected to specific staining procedures. Muscle fibers of extraocular inferior rectus and medial rectus muscle were analyzed in H&E stained slices by CellProfiler (cell image analysis software; Whitehead Institute for Biomedical Research, Cambridge, UK). The total area of the muscle, muscle fiber area, number of muscle fibers and stroma was quantitatively analyzed. Slices of the middle area were stained Picrosirius red to evaluate collagen deposition in the orbital tissue. After dewaxing, the sections were stained with Weigert’s hematoxylin solution (10 minutes, Merck), followed by Picrosirius Red solution (60 minutes, Sirius red solved in 1,3% picric acid - Siriusred, Waldeck-Chroma). Picrosirius red staining of the slices was analyzed quantitatively with CellProfiler. Furthermore, consecutive slices of the middle area were stained for UCP-1 (rabbit polyclonal IgG, dilution 1:1000, Alpha Diagnostics, #UCP11-A), CD31 (PECAM-1, rabbit polyclonal IgG, dilution 1:200, Cell Signaling, #77699), F4/80 (EMR-1, Rat polyclonal IgG, dilution 1:100, Abd Serotec, #MCA497B), CD3 (Rabbit polyclonal IgG, dilution 1:25; Dako, # A0452) and with HRP-conjugated-Polymer-System in accordance with the manufacturers manual (Zytomed Systems). Total fat area and UCP-1 positive stained adipose tissue were determined with ImageJ. CD31 positive vessels, F4/80 and CD3 positive cells were counted in muscle, peri-neural connective tissue and fat tissue.

The thyroids were formalin fixed and paraffin embedded and sections (1 µm) stained H&E. The total thyroid gland area was analyzed objectively in central representative sections of the whole lobe by using CellProfiler (cell image analysis software; Whitehead Institute for Biomedical Research, Cambridge, UK). Thyroidal morphology was blindly evaluated by two different observers and indexed as hyperactive, hypoactive or normal in comparison to the thyroid morphology of the respective Ctrl mice. Images were generated using an Olympus BX51 microscope.

### Statistical analysis

All results are presented as mean ± s.e.m. Graph Pad software (Prism 7, Software Inc., San Diego, CA, USA) was used for statistical analyses and Z-score analysis. Statistical analysis of all data except MRI data were performed with one-way ANOVA with Bonferroni’s multiple comparison test. Statistical analysis of MRI data was performed with unpaired two-tailed multiple Student’s t test and Mann-Whitney-test. Assumption of normal distribution of values was proven by Kolmogorov-Smirnov test. Statistical analysis of paired groups was performed with two-way ANOVA. P values less than 0.05 (95% confidence interval) were considered significant and are indicated.

## Electronic supplementary material

Supporting information S1-S5 Figure
