# Supplementary material for: Graves’ orbitopathy occurs sex-independently in an autoimmune hyperthyroid mouse model
Source: Sci Rep. 2018 Aug 30;8:13096. doi: 10.1038/s41598-018-31253-4 (PMC6117361; doi:10.1038/s41598-018-31253-4)
Supplement: Supplementary file 1 — Supporting information S1-S5 Figure [file 41598_2018_31253_MOESM1_ESM.docx]

**Graves’ orbitopathy occurs sex-independently in an autoimmune hyperthyroid mouse model**

Anke Schlüter^1,2^, Ulrich Flögel^3^, Salvador Diaz-Cano^4^, Gina-Eva Görtz^1^, Kerstin Stähr^2^, Michael Oeverhaus^5^, Svenja Plöhn^1^, Stefan Mattheis^2^, Lars C Möller^6^, Stephan Lang^2^, Nikolaos E Bechrakis^5^, J Paul Banga^1^, Anja Eckstein^1,5^, Utta Berchner-Pfannschmidt^1*^

**Supporting information S1-S5 Fig**


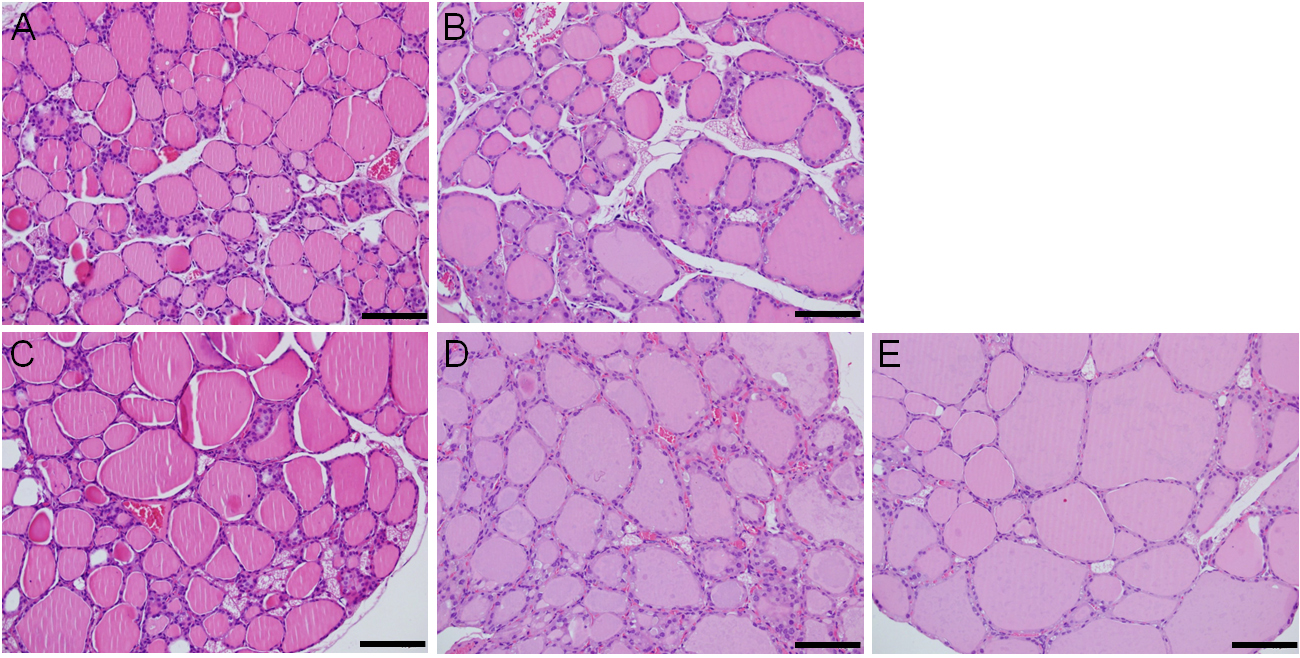


**S1 Fig. Morphology of thyroid glands in GO mouse model.** Thyroid slices of the ß-Gal or hTSHR immunized mice of both sexes were H&E stained and thyroid state was evaluated. Representative images of thyroids are shown. Magnification x20, scale bars represent 100 µm. A Male ß-Gal mouse. B Male hTSHR mouse with hyperactive thyroid state. C Female ß-Gal mouse. D Female hTSHR mouse with hyperactive thyroid state. E Female hTSHR mouse with hypoactive thyroid state. Hyperactive thyroids were characterized by increase of total thyroid size, cuboid cylindrical follicular cells with small amount of colloid, thick follicular epithelium (B, D). Hypoactive thyroids showed thin follicular epithelium and in some follicles the follicular membrane was almost not visible (E).


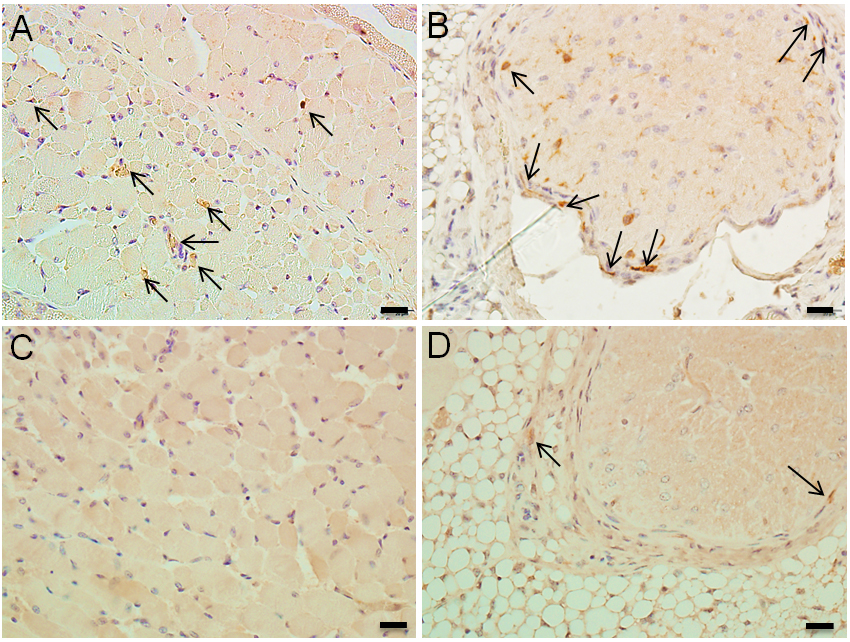


**S2 Fig. Macrophage infiltration in orbital tissues of GO mouse model.** Slices of the middle orbital area were immunohistochemically stained for F4/80 as a marker for macrophages. Representative slices of a male hTSHR (A, B) and ß-Gal (C, D) mouse are shown. Cells positive for F4/80 were counted (arrows) in muscle tissue (A, C) and perinerve connective tissue (B, D). Magnification x40, bars represent 20µm. Number of positive cells was normalized to tissue area as shown in Fig 5A.


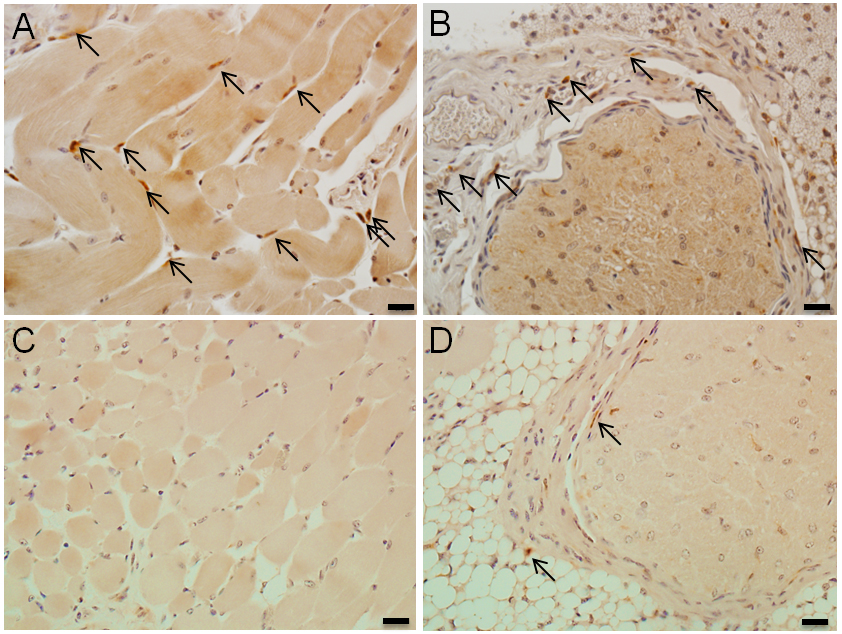


**S3 Fig. T cells infiltration in orbital tissues of GO mouse model.** Slices of the middle orbital area were immunohistochemically stained for CD3 as a marker for T cells. Representative slices of a male hTSHR (A, B) and ß-Gal (C, D) mouse are shown. Cells positive for CD3 were counted (arrows) in muscle tissue (A, C) and perinerve connective tissue (B, D). Magnification x40, bars represent 20µm. Number of positive cells was normalized to tissue area as shown in Fig 5B.


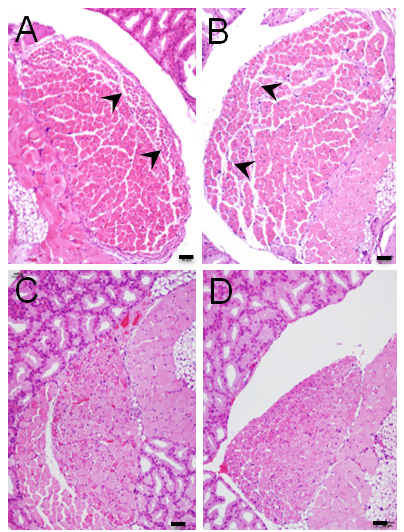


**S4 Fig. Histopathology of extraocular muscles in GO mouse model**. Slices of the middle orbital area were stained H&E. Representative images of extraocular rectus inferior muscle and medial rectus muscle of a female (A, B) and a male (C, D) mouse immunized either with ß-Gal (A, C) or hTSHR A-subunit plasmid (B, D) are shown. (A) Arrows indicates smaller muscle fibers at the edge of control muscle. (B) Arrows indicates enlarged muscle fibers at the edge a muscle from a hTSHR female mouse indicating muscle hypertrophy. (C, D) No such differences in muscle fiber morphology could be detected in male mice. Magnification x4, bars represent 50 µm. The muscle fiber area was quantified and normalized to number of total muscle fiber (Fig 5C).


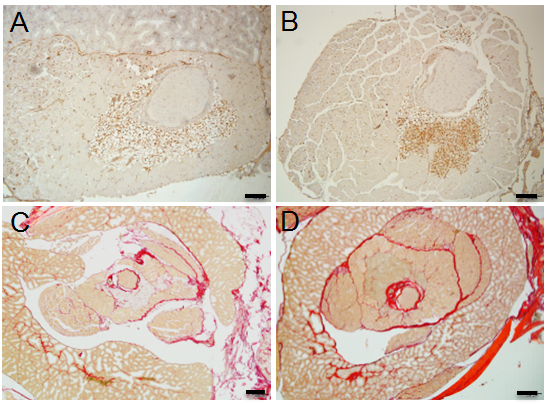


**S5 Fig. Brown adipose tissue and collagen deposition in orbits of GO mouse model**. (A, B) Slices of the middle orbital area were immunohistochemically stained for UCP-1, a marker for browning fat. Representative images of a male ß-Gal (A) or hTSHR (B) mouse are shown. Brown color indicates UCP-1 positive brown fat (BAT). Magnification x10, bars represent 100 µm. Area of brown fat was quantified and normalized to total fat area (Fig 5D). (C, D) Slices were stained Picrosirius red to detect collagens. Representative images of a male ß-Gal (C) or hTSHR (D) mouse are shown. Magnification x4, bars represent 200 µm. Red staining of Picrosirius red was quantified in perineural connective tissue, adipose tissue and extraocular muscle tissue and normalized to total area (Fig 5E).
